# Automated Breast Arterial Calcification Score Is Associated With Cardiovascular Outcomes and Mortality

**DOI:** 10.1016/j.jacadv.2024.101283

**Published:** 2024-09-27

**Authors:** Tara Shrout Allen, Quan M. Bui, Gregory M. Petersen, Richard Mantey, Junhao Wang, Nitesh Nerlekar, Mohammad Eghtedari, Lori B. Daniels

**Affiliations:** a Division of Preventive Medicine, University of California-San Diego, La Jolla, California, USA; b Division of Cardiovascular Medicine, University of California-San Diego, La Jolla, California, USA; c Internal Medicine Residency Program, University of California-San Diego, La Jolla, California, USA; d Research Team, CureMetrix, Inc, La Jolla, California, USA; e Baker Heart and Diabetes Institute, Melbourne, Australia; f Victorian Heart Institute & Monash Health Heart, Monash University, Clayton, Australia; g Department of Radiology, University of California-San Diego, La Jolla, California, USA

**Keywords:** artificial intelligence, personalized risk stratification, prevention, subclinical atherosclerosis, women’s health

## Abstract

**Background:**

Breast arterial calcification (BAC) on mammograms has emerged as a biomarker of women’s cardiovascular disease (CVD) risk, but there is a lack of quantification tools and clinical outcomes studies.

**Objectives:**

This study assessed the association of BAC (both presence and quantity) with CVD outcomes.

**Methods:**

This single-center, retrospective study included women with a screening mammogram from 2007 to 2016. BAC was quantified using an artificial intelligence-generated score, which was assessed as both a binary and continuous variable. Regression analyses evaluated the association between BAC and mortality and a composite of acute myocardial infarction, heart failure, stroke, and mortality. Analyses were adjusted for age, race, diabetes, smoking, blood pressure, cholesterol, and history of CVD and chronic kidney disease.

**Results:**

A total of 18,092 women were included in this study (mean age 56.8 ± 11.0 years; diabetes [13%], hypertension [36%], hyperlipidemia [40%], and smoking [5%]). BAC was present in 4,223 (23%). Over a median follow-up of 6 years, death occurred in 7.8% and 2.3% of women with and without BAC, respectively. The composite occurred in 12.4% and 4.3% of women with and without BAC, respectively. Compared to those without, women with BAC had adjusted HRs of 1.49 (95% CI: 1.33-1.67) for mortality and 1.56 (95% CI: 1.41-1.72) for the composite. Each 10-point increase in the BAC score was associated with higher risk of mortality (HR: 1.08 [95% CI: 1.06-1.11]) and the composite (HR: 1.08 [95% CI: 1.06-1.10]). BAC was especially predictive of future events among younger women.

**Conclusions:**

BAC is independently associated with mortality and CVD, especially among younger women. Measurement of BAC beyond presence adds incremental risk stratification. Quantifying BAC using an artificial intelligence algorithm is feasible, clinically relevant, and may improve personalized CVD risk stratification.

Cardiovascular disease (CVD) remains the leading cause of death in women despite significant advances in cardiovascular diagnostics and treatments.^1^ Delays in diagnosis and treatment, as well as undertreatment, contribute to morbidity and mortality.^2^ This is further exacerbated by under-representation of women in cardiovascular clinical trials and lack of sex-specific screening tools.^3^ Efficient and effective methods to broadly screen women for CVD risk are sorely needed.

Breast arterial calcification (BAC), an incidental finding on mammograms, has emerged as a sex-specific biomarker for atherosclerotic cardiovascular disease (ASCVD) that offers the potential for personalized risk stratification.^4^ The prevalence of mammographic BAC increases with age, occurring in 10% of women at age 40 but in up to 50% by age 80 years.^5^, ^6^, ^7^ In semiquantitative analysis using radiologist assessments, high-grade or severe BAC was rare in younger women, but approached 14% by age 70 years.^8^

Gleaning information from an imaging study beyond its original intent is not new; analogous to BAC on mammography is coronary artery calcifications (CACs) seen on chest computed tomography obtained for noncardiac purposes.^9^ BAC has tremendous appeal for cardiovascular risk stratification because it is noninvasive, comes at no additional cost or radiation, and the majority of women over the age of 40 years already undergo annual screening mammography for breast cancer.^10^

Multiple studies have found significant associations between the presence of BAC and prevalent CVD.^4^ It is postulated that BAC represents lifetime exposure to risk factors related to arterial stiffening, which increases the risk of CVD through both coronary and noncoronary mechanisms (ie, heart failure [HF] and stroke).^11^ However, routine clinical use of BAC has not been adopted due to a lack of outcomes studies as well as technological challenges in measuring and reporting BAC.^4^ Currently, there is no consensus recommendation on the inclusion or standardized reporting of BAC, and American College of Radiology guidelines on breast imaging classifies reporting of vascular calcifications as optional.^12^^,^^13^ However, in 2023, the Canadian Society of Breast Imaging took a progressive stance, advocating for standardized reporting of BAC in mammogram reports.^14^

Moreover, most BAC studies are limited to the binary presence or absence of BAC, and thus are blind to the severity or burden of BAC. Few studies measure or categorize BAC by severity and there is significant heterogeneity in classification.^7^ The purpose of this study was to evaluate not only the association of BAC presence with CVD risk factors and hard clinical outcomes in a large population but also to validate the utility of a novel automated, artificial intelligence (AI) algorithm for personalized BAC quantification.

## Methods

### Study population

This single-center retrospective study included women between the ages of 40 and 90 years who underwent screening digital mammography between 2007 and 2016 at the University of California-San Diego Health. For each subject, only the index mammogram was analyzed. All protocols were approved by the Institutional Review Board (IRB #170154).

### Evaluation of BAC

BAC was quantified using a validated, proprietary investigational software (cmAngio, CureMetrix) based on a deep neural, AI network, and previously trained with an 80:20 split using over 34,000 2D full-field digital mammograms and digital breast tomosynthesis mammograms obtained from multiple sites across 13 health care facilities in Australia, Brazil, and the United States (not including University of California San Diego Health).

As a standard, 4 full-field digital mammograms or digital breast tomosynthesis images from each participant were used. The software cmAngio assesses screening mammography images and feeds them through the deep learning model to identify regions of interest within the breast. These regions correspond to areas that the algorithm suspects to have a high probability of BAC. From these identified regions, local and global imaging features such as density, contrast, and other physical dimensions are combined to determine the presence and severity of BAC. This process is applied to each of the 4 standard screening mammography images. Following these calculations, each image is assigned a score between 0 and 100 corresponding to the severity of the BAC finding(s), with 0 representing no BAC and 100 representing the highest percentile of BAC. To balance the algorithm’s false positive and false negative rate, all image-level scores less than 5 are floored to 0. The patient-level score (or BAC score) is the mean of the threshold image-level scores across all 4 views. As such, BAC presence was defined as a mean BAC score ≥5. BAC was evaluated as a binary variable (presence vs absence), continuous variable (BAC score 0-100), and quartile groups (first-fourth). Scores were distributed by severity into the following groups: first quartile [score 1-25], second quartile [score 26-50], third quartile [score 51-75], and fourth quartile [score 76-100].

During development, each case was reviewed by 2 of 11 Mammography Quality Standards Act-certified radiologists. The performance of the software for detecting BAC, as assessed by area under the receiver operating characteristic curve was 0.98, with a sensitivity of 94% and a specificity of 96%. The software is cleared for BAC detection by the Food and Drug Administration and has been deployed in investigational clinical settings with Institutional Review Board approval.

### Clinical data and outcomes

All clinical data including baseline characteristics and outcomes were collected using electronic health records (EHRs) and International Classification of Diseases (ICD)-10 codes, which are provided in Supplemental Table 1. All incident diagnoses occurred at least 6 months after the index mammogram and until death or the censoring date of December 31, 2020. The primary outcome was all-cause mortality. Secondary outcomes included acute myocardial infarction (MI), HF, stroke, and a cardiovascular composite outcome (MI, HF, stroke, and mortality). Stroke (cerebrovascular disease) included ischemic and hemorrhagic stroke. Those with baseline MI, HF, or stroke were excluded from the relevant outcome analyses, including the composite outcome. Additionally, in a sensitivity analysis, all participants with baseline ASCVD were excluded to reassess the associations. ASCVD was defined by the following ICD-10 diagnoses: ASCVD, coronary artery disease (CAD), peripheral arterial disease (PAD), HF, and/or cerebrovascular disease.

### Analyses and statistical methods

Continuous variables were reported either as mean with standard deviation or as median with interquartile range as appropriate based on normality of distribution assessed by Shapiro-Wilk test. Categorical variables were expressed as counts with percentages. Variables were compared using the unpaired Student *t*-test, Mann-Whitney test, and Fisher’s exact test, as appropriate. Proportional hazards assumptions were tested for all outcomes to verify modeling assumption. Furthermore, Schoenfeld residual plots were generated for confirmation. Kaplan-Meier survival curves (plotted with 95% CIs), cumulative incidence plots (as appropriate), and Cox proportional hazards regression analyses were used to determine associations between BAC (as a binary and continuous variable) and clinical outcomes, while adjusting for variables at the time of mammogram (age, race/ethnicity, smoking status, systolic blood pressure, diastolic blood pressure, total cholesterol, low-density lipoprotein [LDL] cholesterol, diabetes mellitus, and a history of CVD or chronic kidney disease [CKD]).

Age was continuous and measured in years. Smoking status was categorical and defined as current, former, never, or unknown. Systolic and diastolic blood pressures were continuous and measured in mm Hg. Total cholesterol and LDL cholesterol were continuous and measured in mg/dL. For other covariates, diabetes mellitus and CKD were defined by the associated ICD-10 code (Supplemental Table 1). CVD was defined as an ICD-10 code for any of the following: ASCVD, MI, CAD, HF, and/or stroke (cerebrovascular disease). For those without covariate data from the time of the index mammogram, imputation was performed to account for these missing data. Data were imputed by training a nearest neighbor multiple-imputation model in Python to predict missing variables using the 10 nearest neighbors based on the collected diagnosis codes, age, ethnicity, smoking status, blood pressure (systolic and diastolic), and cholesterol (total and LDL).

Forest plots were created to assess the association between BAC and outcomes, stratified by subgroups of baseline characteristics. Tails represent 95% CIs. All reported *P* values were 2-sided with a value of <0.05 considered statistically significant. Statistical analyses and figures were completed using Python 3.11.5 with packages including Pandas 2.1.0 and SciPy 1.11.2.

## Results

### Study population

**Figure 1:**
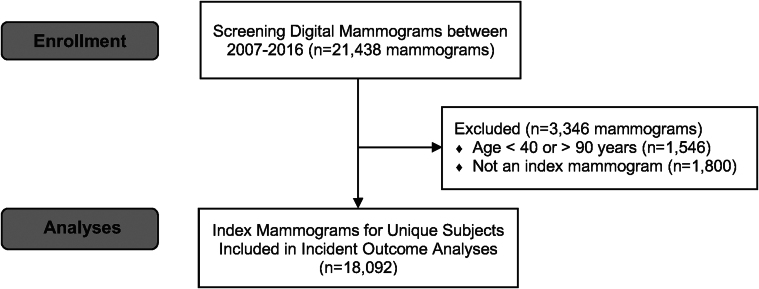
**Participant Flow Diagram** After exclusions for age and non-index mammograms, there were 18,092 unique women with index mammograms included in this study.

There were 21,438 screening mammograms obtained between 2007 and 2016. Of these, 1,546 were excluded for age and 1,800 were excluded for not being the index study. Therefore, 18,092 women with index mammograms were included in the study (Figure 1). Among the 18,092 women included, the mean age was 56.8 ± 11.0 years with prevalent CVD risk factors of diabetes (13%), hypertension (36%), and hyperlipidemia (40%) (Table 1). BAC was present in 4,223 (23%). BAC was more prevalent among women who were older, Black or Hispanic, diabetic, hypertensive, with a history of ASCVD or CKD, and taking statins and/or antihypertensive medications. BAC was less prevalent in current smokers. Among those with BAC, the median score was 15 (IQR: 4, 50). Scores were distributed by severity into the following quartile groups: first quartile [score 1-25], n = 2,552 (60.4%); second quartile [score 26-50], n = 643 (15.2%); third quartile [score 51-75], n = 509 (12.1%); and fourth quartile [score 76-100], n = 519 (12.3%). Correspondingly, those with a higher BAC score were more likely to be older, diabetic, hypertensive, having a history of CVD, CKD or hyperlipidemia, and taking statin and antihypertensive medications. (Supplemental Table 2). Additionally, details on imputation and missing covariate data are presented in Supplemental Table 3.

**Table 1:** Baseline Participant Characteristics by Presence of Breast Arterial Calcification

	Total (n = 18,092)	BAC Present (n = 4,223; 23%)	BAC Absent (n = 13,869; 77%)	*P* Value
Age, y	56.8 ± 11.4	65.2 ± 11.6	54.2 ± 10.0	<0.001
Race/ethnicity				
Caucasian	11,319 (62.6)	2,617 (62.0)	8,702 (62.7)	0.38
Black/African American	907 (5.0)	241 (5.7)	666 (4.8)	0.02
Hispanic/Latino	1,694 (9.4)	455 (10.8)	1,239 (8.9)	<0.001
Asian/Pacific Islander	2,321 (12.8)	496 (11.8)	1,825 (13.2)	0.02
Other	1,851 (10.2)	414 (9.8)	1,437 (10.4)	0.31
Diabetes	2,267 (12.5)	730 (17.3)	1,537 (11.1)	<0.001
Hypertension	6,529 (36.1)	2,179 (51.6)	4,350 (31.4)	<0.001
Hyperlipidemia	7,256 (40.1)	2,071 (49.0)	5,185 (37.4)	<0.001
History of CVD	874 (4.8)	424 (10.0)	450 (3.2)	<0.001
History of CKD	802 (4.4)	358 (8.48)	444 (3.2)	<0.001
Current smoking	834 (4.6)	134 (3.17)	700 (5.1)	<0.001
Never smokers	9,245 (51.1)	2,046 (48.5)	7,199 (52.6)	<0.001
Systolic blood pressure, mm Hg	123 (21)	128 (20)	122 (20)	<0.001
Total cholesterol, mg/dL	198 (52)	194 (53)	199 (51)	<0.001
Statin use	3,947 (21.8)	1,430 (33.9)	2,517 (18.1)	<0.001
Antihypertensive use	3,498 (19.3)	1,313 (31.1)	2,185 (15.8)	<0.001

Values are mean ± SD, n (%), or median (IQR).

BAC = breast arterial calcification; CKD = chronic kidney disease; CVD = cardiovascular disease.

### Clinical outcomes

**Figure 2:**
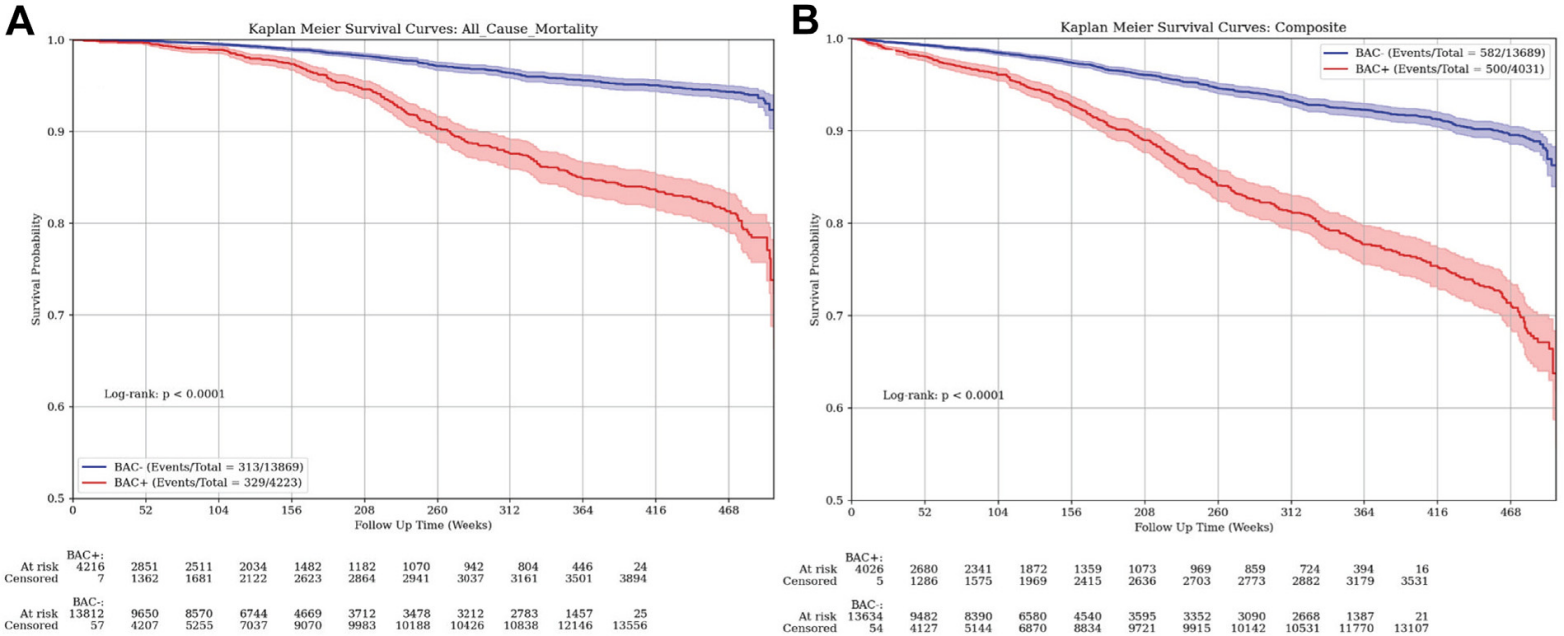
Kaplan-Meier Plots for Mortality and Composite Outcome by Breast Arterial Calcification Presence Risk for (A) mortality, and (B) the cardiovascular composite outcome significantly varied by the presence of breast arterial calcification (*P* < 0.001 for each). The composite outcome included acute myocardial infarction, heart failure, stroke, and mortality. Time points of 208 weeks and 468 weeks are indicative of approximately 4 years and 9 years, respectively. BAC = breast arterial calcification; BAC+ = presence of BAC; BAC− = absence of BAC.

Over a median follow-up for mortality of 4.8 years (IQR: 4.2 years), there were 329 deaths in those with BAC (7.8%) and 313 deaths in those without BAC (2.3%) (*P* < 0.001) (Table 2). Over a median follow-up for the composite outcome of 4.3 years (IQR: 4.3 years), there were 500 events in those with BAC (12.4%) and 582 events in those without BAC (4.3%) (*P* < 0.001). Stroke, MI, and HF were more frequently observed in those with BAC present, although the competing risk of death precludes statistical comparison. Kaplan-Meier Plots for mortality and the composite outcome are shown in Figure 2, which demonstrate a significantly increased risk of outcomes in those with BAC (*P* < 0.001 for each). Additionally, for HF, over a median follow-up of 3.0 years (IQR: 4.6 years), there were 154 events in those with BAC (3.7%) and 144 events in those without BAC (1.0%) (*P* < 0.001). For MI, over a median follow-up of 3.3 years (IQR: 3.9 years), there were 36 events in those with BAC (0.9%) and 47 events in those without BAC (0.3%) (*P* < 0.001). Lastly, for stroke, over a median follow-up of 3.0 years (IQR: 4.7 years), there were 110 events in those with BAC (2.7%) and 149 events in those without BAC (1.1%) (*P* < 0.001). Cumulative incidence plots for individual outcomes of stroke, MI, and HF are shown in Supplemental Figure 1, which also demonstrate significantly increased risk in those with BAC (*P* < 0.001 for each outcome).

**Table 2:** Clinical Outcomes by Breast Arterial Calcification Presence

	Total (N = 18,092)		BAC Present (n = 4,223)		BAC Absent (n = 13,869)		*P* Value
Myocardial infarction	18,051	83 (0.5%)	4,204	36 (0.9%)	13,847	47 (0.3%)	
Heart failure	17,911	298 (1.7%)	4,119	154 (3.7%)	13,792	144 (1.0%)	
Stroke	17,914	259 (1.5%)	4,138	110 (2.7%)	13,776	149 (1.1%)	
Mortality	18,092	642 (3.6%)	4,223	329 (7.8%)	13,869	313 (2.3%)	<0.001
Composite outcome^a^	17,720	1,082 (6.1%)	4,031	500 (12.4%)	13,689	582 (4.3%)	<0.001

Values are N or n (%).

Abbreviations as in Table 1.

a The cardiovascular composite outcome included acute myocardial infarction, heart failure, stroke, and mortality.

In multivariable analysis, women with BAC present had a significantly higher risk of mortality (adjusted HR [aHR]: 1.49 [95% CI: 1.33-1.68], *P* < 0.001) and the composite outcome (aHR: 1.57 [95% CI: 1.42-1.74], *P* < 0.001), compared to those without BAC (Table 3). Exclusion of those prescribed statin therapy (n = 3,947) did not materially affect the results: mortality aHR 1.45 (95% CI: 1.29-1.63), *P* < 0.001 and the composite outcome aHR 1.53 (95% CI: 1.38-1.69), *P* < 0.001 (Table 3). After excluding those with any baseline ASCVD, results were essentially unchanged (Table 3). For example, for the morality outcome, exclusion of 758 participants with baseline ASCVD still led to a significant difference (aHR: 1.44 [95% CI: 1.28-1.62]; *P* < 0.001). For the composite outcome, exclusion of those with baseline ASCVD, CAD, and PAD (n = 399) did not significantly alter the results (aHR: 1.50 [95% CI: 1.35-1.66]; *P* < 0.001) (Table 3).

**Table 3:** Association of Breast Arterial Calcification Presence and Clinical Outcomes

	Mortality HR (95% CI)	*P* Value	Composite Outcome^a^ HR (95% CI)	*P* Value
Among all participants	(n = 642/18,092)		(n = 1,082/17,720)	
Model 1	1.70 (1.52-1.90)	<0.001	1.92 (1.74-2.11)	<0.001
Model 2	1.58 (1.41-1.77)	<0.001	1.67 (1.51-1.84)	<0.001
Model 3	1.49 (1.33-1.68)	<0.001	1.57 (1.42-1.74)	<0.001
Excluding those prescribed statins	(n = 400/14,145)		(n = 739/14,145)	
Model 3	1.45 (1.29-1.63)	<0.001	1.53 (1.38-1.69)	<0.001
Excluding those with baseline ASCVD^b^	(n = 565/17,334)		(n = 1,025/17,321)	
Model 3	1.44 (1.28-1.62)	<0.001	1.50 (1.35-1.66)	<0.001

ASCVD = atherosclerotic cardiovascular disease.

a Composite outcome: acute myocardial infarction, heart failure, stroke, and mortality.

b An additional 758 participants with any baseline ASCVD were excluded for the mortality outcome and an additional 399 participants with specific baseline conditions not already accounted for were excluded for the composite outcome. Model 1: unadjusted. Model 2: adjusted for age and race/ethnicity. Model 3: adjusted for age, race/ethnicity, systolic blood pressure, diastolic blood pressure, diabetes, total cholesterol, low-density lipoprotein cholesterol, history of cardiovascular disease, history of chronic kidney disease, and smoking status.

When BAC was quantified and analyzed as a continuous score, each 10-point increase in the BAC score was significantly and independently associated with higher risk for adverse outcomes: mortality (aHR: 1.08 [95% CI: 1.06-1.11]; *P* < 0.001)and composite outcome (aHR: 1.08 [95% CI: 1.06-1.10]; *P* < 0.001) (Table 4). After excluding those on statin therapy, results again were unchanged: mortality (aHR: 1.01 [95% CI: 1.007-1.013]; *P* < 0.001) and the composite outcome (aHR: 1.01 [95% CI: 1.008-1.013]; *P* < 0.001). After excluding those with baseline ASCVD, results again remained significant for both mortality (aHR: 1.01 [95% CI: 1.006-1.011]; *P* < 0.001) and the composite outcome (aHR: 1.01 [95% CI: 1.007-1.011]; *P* < 0.001) (Table 4).

**Table 4:** Association of the Breast Arterial Calcification Score and Clinical Outcomes

	Mortality aHR (95% CI)	*P* Value	Composite Outcome^a^ aHR (95% CI)	*P* Value
Among all participants	(n = 642/18,092)		(n = 1,082/17,720)	
BAC negative, n = 13,869	Referent	--	Referent	--
Per 10-point BAC score increase	1.08 (1.06-1.11)	**<0.001**	1.08 (1.06-1.10)	**<0.001**
First quartile [score 1-25], n = 2,552	1.22 (1.06-1.41)	**0.006**	1.26 (1.11-1.43)	**<0.001**
Second quartile [score 26-50], n = 643	1.44 (1.13-1.85)	**0.004**	1.74 (1.42-2.13)	**<0.001**
Third quartile [score 51-75], n = 509	1.69 (1.33-2.14)	**<0.001**	1.83 (1.49-2.25)	**<0.001**
Fourth quartile [score 76-100], n = 519	2.27 (1.81-2.85)	**<0.001**	2.30 (1.88-2.82)	**<0.001**
Excluding those prescribed statins	(n = 400/14,145)		(n = 739/14,145)	
BAC negative, n = 11,352	Referent	--	Referent	--
Per 10-point BAC score increase	1.01 (1.007-1.013)	**<0.001**	1.01 (1.008-1.013)	**<0.001**
First quartile [score 1-25], n = 1,838	1.27 (1.07-1.51)	**0.007**	1.25 (1.07-1.46)	**0.006**
Second quartile [score 26-50], n = 390	1.48 (1.07-2.06)	**0.018**	1.72 (1.31-2.27)	**<0.001**
Third quartile [score 51-75], n = 295	1.58 (1.13-2.19)	**0.007**	1.69 (1.26-2.25)	**<0.001**
Fourth quartile [score 76-100], n = 270	2.53 (1.81-3.53)	**<0.001**	2.61 (1.97-3.47)	**<0.001**
Excluding those with baseline ASCVD^b^	(n = 565/17,428)		(n = 1,025/17,428)	
BAC negative, n = 13,540	Referent	--	Referent	--
Per 10-point BAC score increase	1.01 (1.006-1.011)	**<0.001**	1.01 (1.007-1.011)	**<0.001**
First quartile [score 1-25], n = 2,414	1.21 (1.05-1.40)	**0.007**	1.15 (0.97-1.36)	0.111
Second quartile [score 26-50], n = 593	1.39 (1.09-1.78)	**0.008**	1.34 (0.98-1.83)	0.071
Third quartile [score 51-75], n = 459	1.59 (1.26-2.01)	**<0.001**	1.62 (1.15-2.29)	**0.006**
Fourth quartile [score 76-100], n = 422	2.20 (1.75-2.75)	**<0.001**	2.14 (1.53-3.00)	**<0.001**

aHR = adjusted HR; other abbreviation as in Table 3.

a Composite outcome: acute myocardial infarction, heart failure, stroke, and mortality.

b An additional 758 participants with any baseline ASCVD were excluded for the mortality outcome and an additional 399 participants with specific baseline conditions not already accounted for were excluded for the composite outcome. All data from the multivariable-adjusted model (Model 3), which adjusted for age, race/ethnicity, systolic blood pressure, diastolic blood pressure, diabetes, total cholesterol, low-density lipoprotein cholesterol, history of CVD, history of chronic kidney disease, and smoking status.

**Figure 3:**
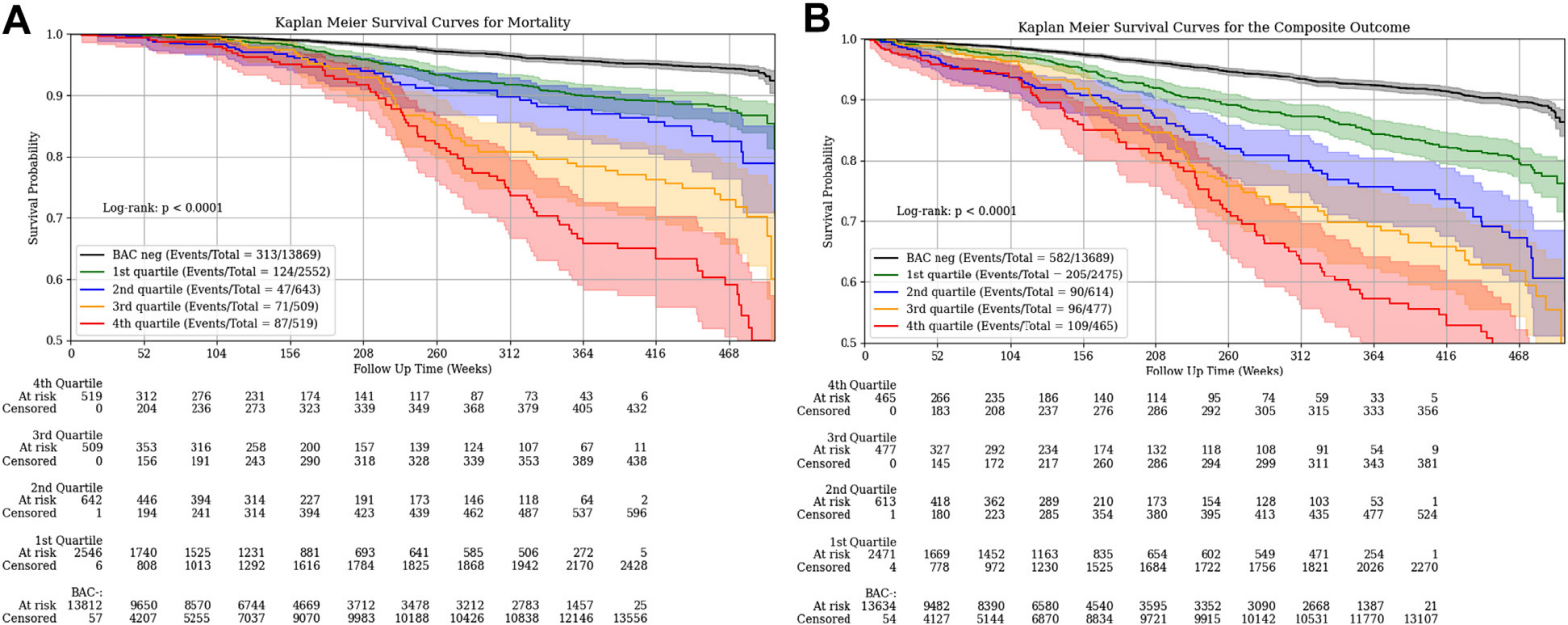
Kaplan-Meier Plots for Mortality and Composite Outcome by Breast Arterial Calcification Score Quartiles Risk for (A) mortality, and (B) the cardiovascular composite outcome significantly varied by the quantified breast arterial calcification score quartile (log-rank *P* < 0.001 for each). The composite outcome included acute myocardial infarction, heart failure, stroke, and mortality. Time points of 208 weeks and 468 weeks are indicative of approximately 4 years and 9 years, respectively. BAC = breast arterial calcification; BAC+ = presence of BAC; BAC− = absence of BAC.

When assessed by BAC score quartiles, there was a significantly higher risk in a consistently graded manner for both mortality and the composite outcome (Figure 3), even after adjustment for cardiovascular risk factors (Table 4). After excluding those on statin therapy, there were no significant differences (Table 4). After excluding those with baseline ASCVD, similar results were seen for mortality, though for the composite outcome, the graded association only reached statistical significance starting with the third quartile (Table 4).

Similar associations were also seen for HF and stroke, though results for MI (only 83 incident events) did not reach statistical significance (Supplemental Figure 2, Supplemental Tables 4 and 5) of BAC. Time points of 208 weeks and 468 weeks are indicative of approximately 4 years and 9 years, respectively.

### Breast arterial calcification and clinical outcomes among subgroup

**Figure 4:**
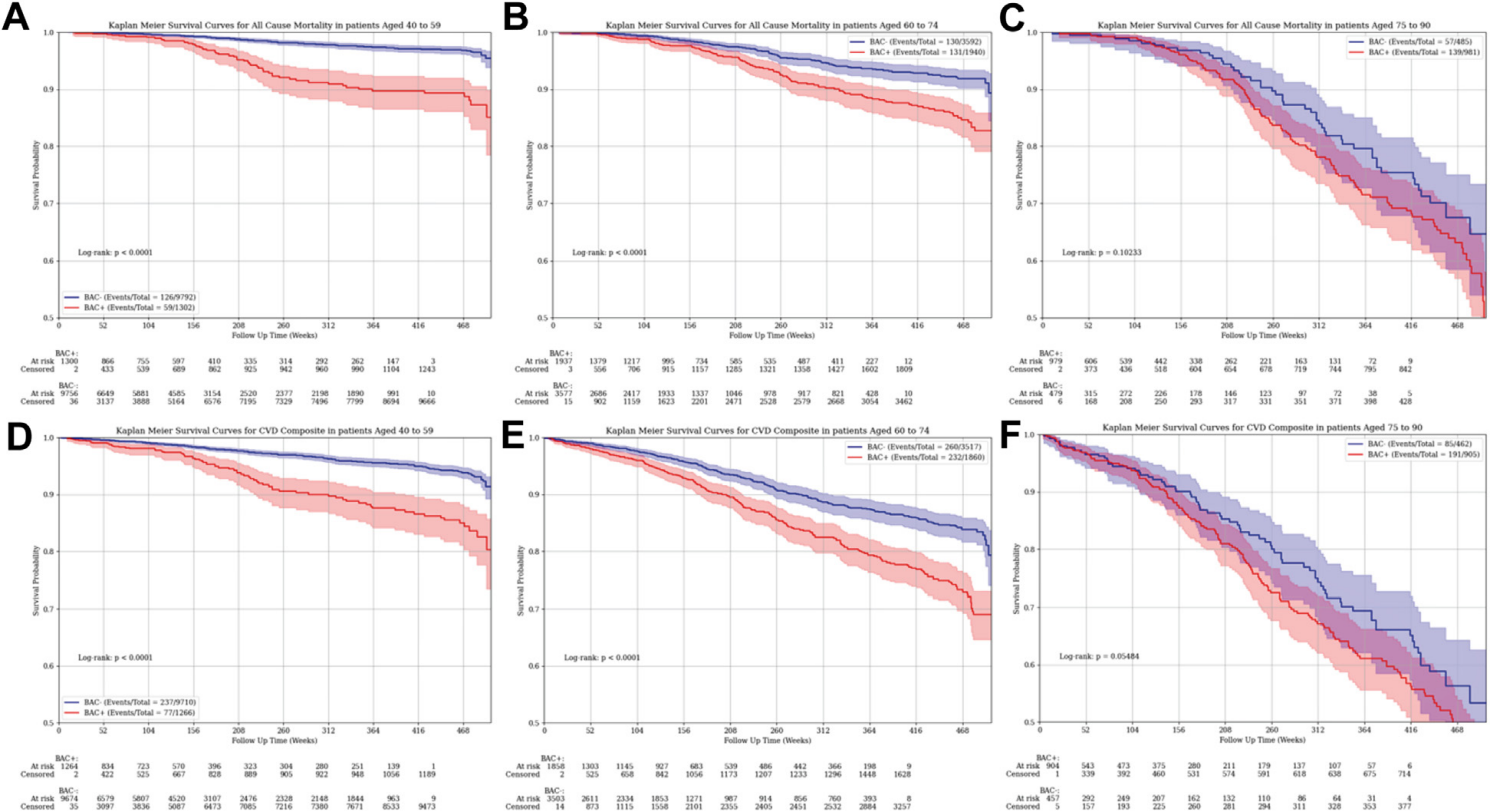
Association of Breast Arterial Calcification and Mortality and Cardiovascular Composite Outcome Stratified by Age Groups Risk for mortality (A to C) and the cardiovascular composite outcome (D to F) by breast arterial calcification (BAC) presence/absence. Risk for both outcomes significantly varied by BAC status among women aged 40 to 59 years (A and D) and those aged 60 to 74 years (B and E) (*P* < 0.001 for each); however, among women aged 75 to 90 years (C and F), there was no significant difference in risk for either outcome by BAC status. The composite outcome included acute myocardial infarction, heart failure, stroke, and mortality. Time points of 208 weeks and 468 weeks are indicative of approximately 4 years and 9 years, respectively. BAC = breast arterial calcification; BAC+ = presence of BAC; BAC− = absence of BAC.

BAC prediction for mortality and the composite cardiovascular outcome significantly varied by age, systolic blood pressure, total cholesterol, LDL cholesterol, smoking, and diabetes (*P* interaction terms <0.001 for each). Additionally, prediction significantly varied by history of CVD for mortality (*P* interaction term <0.001) and the composite outcome (*P* interaction term 0.009). While prediction also significantly varied by history of CKD for mortality (*P* interaction term 0.004), it did not for the composite outcome (*P* interaction term 0.16). Kaplan-Meier plots for mortality and the composite outcome stratified by age groups (Figure 4) demonstrate a significant separation of curves for women aged 40 to 59 and 60 to 74 years of age (*P* < 0.001) but not for those aged 75 to 90 years (morality, *P* = 0.10; composite, *P* = 0.05).

**Figure 5:**
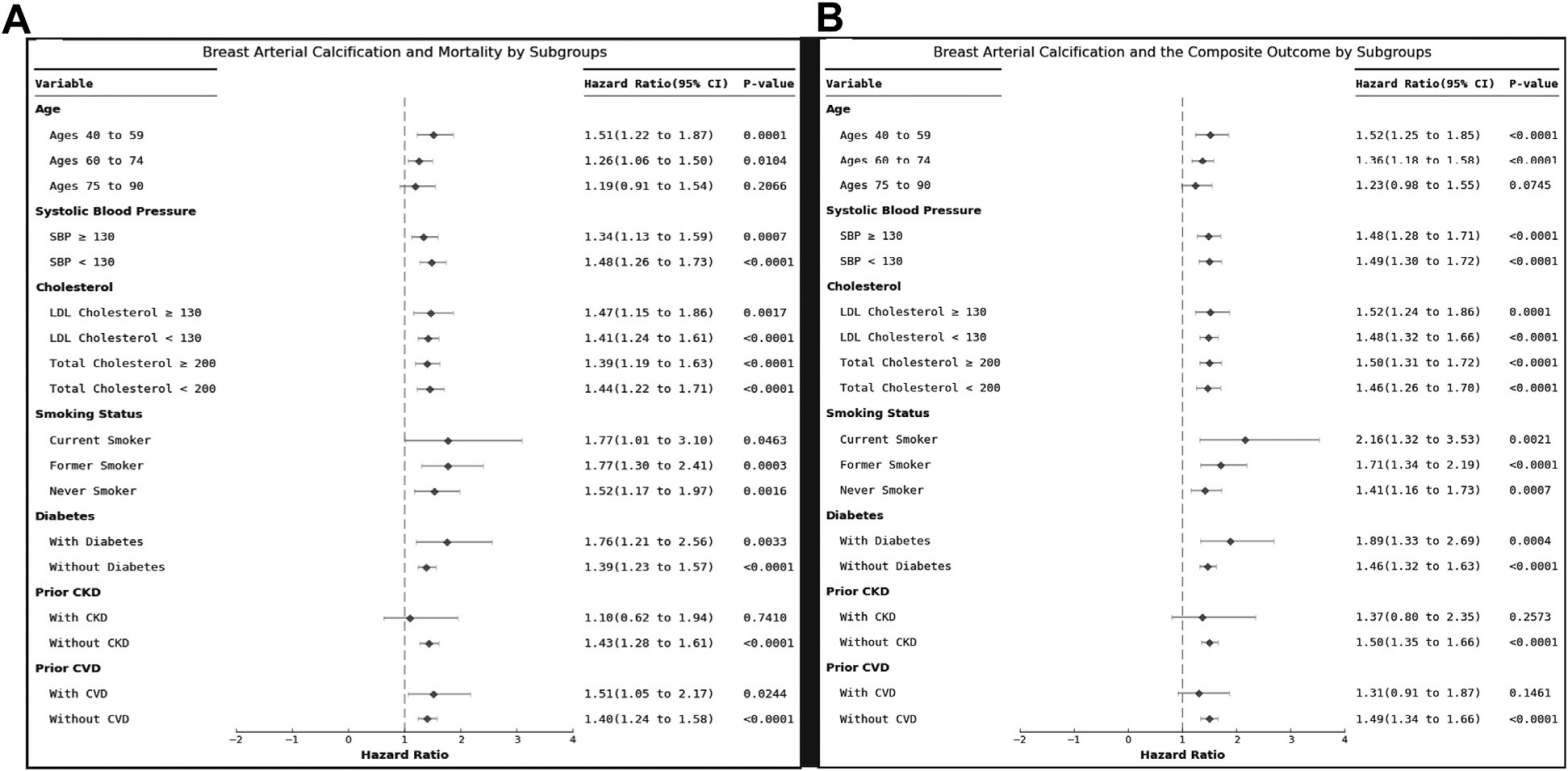
Association of Breast Arterial Calcification and Mortality and Cardiovascular Composite Stratified by Baseline Characteristics Adjusted HRs for (A) mortality and (B) the cardiovascular composite outcome by breast arterial calcification (BAC) presence vs absence are presented. The composite outcome included acute myocardial infarction, heart failure, stroke, and mortality. HRs presented were adjusted for age, race/ethnicity, systolic blood pressure, diastolic blood pressure, diabetes, total cholesterol, low-density lipoprotein cholesterol, smoking status, and history of cardiovascular disease, history of chronic kidney disease. BAC = breast arterial calcification; BAC+ = presence of BAC; BAC− = absence of BAC.

Forest plots demonstrating aHRs for outcomes by stratification of baseline characteristics are shown in Figure 5. When stratified by age groups, and after accounting for traditional risk factors, those in the youngest age group of 40 to 59 years had the highest residual risk associated with BAC (mortality: aHR: 1.51; 95% CI: 1.22-1.87; composite outcome: aHR: 1.52; 95% CI: 1.25-1.85). There remained significantly increased risk associated with BAC beyond traditional risk factors for women aged 60 to 74 years (mortality: aHR: 1.26; 95% CI: 1.06-1.50; composite outcome: aHR: 1.36; 95% CI: 1.18-1.58) but not among those aged 75 to 90 years (mortality: aHR: 1.19; 95% CI: 0.91-1.54; composite outcome: aHR: 1.23; 95% CI: 0.98-1.55). When stratified by other baseline characteristics, including systolic blood pressure and diabetes, the association between BAC and future cardiovascular events remained robust, even after accounting for traditional risk factors (Figure 5).

## Discussion

In this large, retrospective study, both the presence and quantity of BAC were significantly associated with all-cause mortality and the CVD composite outcome, even after adjusting for established cardiovascular risk factors. The prevalence of BAC was 23%, which constitutes a substantial proportion of women (mean age of 56.8 years) undergoing routine screening mammography. To our knowledge, this is the first study to demonstrate a significant, independent relationship between a quantitative BAC score and all-cause mortality or a CVD composite outcome. Indeed, each 10-point increase as well as sequential quartiles of the BAC score were significantly associated with higher risk of mortality and adverse cardiovascular outcomes, highlighting the potential utility of BAC quantification for personalized risk assessment (Central Illustration).

**Central Illustration:**
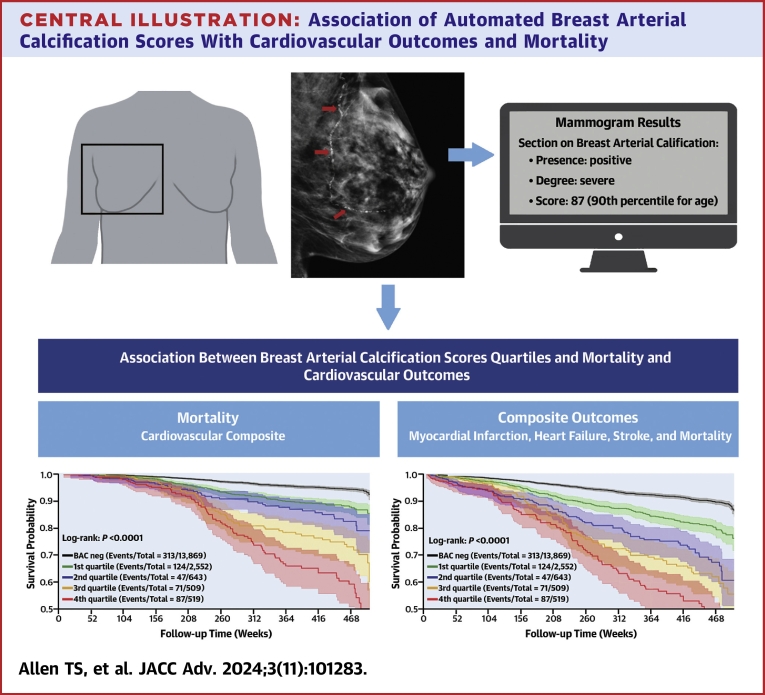
**Association of Automated Breast Arterial Calcification Scores With Cardiovascular Outcomes andMortality**

Prior studies have evaluated the association of BAC using a binary or a semiquantitative approach (such as absence, slight, moderate, and severe intensity) with CVD outcomes.^4^^,^^15^ In the present study, BAC was quantified using an automated method driven by a trained deep neural AI network, recently validated with high diagnostic performance.^16^ Other machine learning techniques have been developed for BAC quantification, including a densitometry method, and have been validated prospectively.^17^ Such studies have assessed methods of BAC quantification, though await association with clinical outcomes.^18^, ^19^, ^20^ The findings in our study support the efficacy of assessing both BAC presence and a quantitative BAC score to improve risk assessment for mortality and CVD outcomes in women undergoing screening mammography. With the advent of AI in medical imaging, automated, quantitative BAC assessment may facilitate seamless integration into clinical workflow and allow personalized risk assessment.

Importantly, this study also demonstrates the association of BAC with CVD outcomes and mortality even among subgroups not already known to be “high risk,” including younger women, nonsmokers, and those without diabetes, hypertension, hyperlipidemia, CKD, or known CVD. We found that BAC was most predictive of future events among those in the youngest age group of 40 to 59 years, though BAC was also an independent predictor among women ages 60 to 74 years. Our results are concordant with those of Minssen et al^21^ who found that the diagnostic accuracy (∼84%) for BAC with CACs was the highest in patients under the age of 60 years. Results from this study and others suggest that BAC may develop at an earlier age than other traditional cardiovascular risk factors, and thus could serve as an early biomarker of underlying ASCVD risk.^22^ These findings are important since they suggest that early risk stratification with BAC in younger women may help identify new candidates for lifestyle modification and preventative therapies and may ultimately help improve their outcomes. Moreover, we find that quantifying BAC allows us to better stratify risk with a graded association for both mortality and the composite outcome. Thus, simply reporting BAC presence or absence is insufficient and leaves valuable information underutilized.

Even with engagement from cardiologists and patients, the success of BAC implementation hinges on buy-in and education of the radiology community. A survey of the members of the Society of Breast Imaging found that 85% were aware of the association of BAC with CVD, but only 15% routinely included BAC data on mammogram reports.^6^ One of the major barriers to universal BAC reporting is the lack of radiology society guidelines on reporting and appropriate use of BAC.^6^^,^^9^ Automated quantification and reporting methods for BAC will be critical to ensure that the current radiology workflow is not compromised.^11^ Therefore, it will be important for cardiologists to advise and collaborate with the breast imaging community to develop clear BAC reporting guidelines and apply automated quantification tools into clinical workflow.

If the development and implementation of BAC can follow a similar pathway as CACs, BAC may someday be used to improve CVD risk stratification beyond current tools such as the pooled cohort equation, the ASCVD Risk Score, and the Framingham Risk Score. Reclassification of risk will help identify those who will benefit from more aggressive lifestyle modifications and medical therapy (ie, statins, antihypertensives).

Recently, the MINERVA (Multiethnic Study of Breast Arterial Calcium Gradation and Cardiovascular Disease) demonstrated that presence of BAC conferred additional risk at every category (ie, low, medium, and high risk) of the pooled cohort equation.^17^ While our study does not address CVD risk discrimination modeling, we demonstrate that BAC can reliably be quantified using a novel AI algorithm and is independently associated with mortality and various CVD outcomes, which is a crucial and impactful step in this field. Future work will assess whether BAC scores can improve existing risk assessments for CVD outcomes, especially among women of intermediate ASCVD risk to guide initiation of preventive measures, such as statins, similar to CAC scores as suggested in the 2018 American College of Cardiology/American Heart Association Cholesterol Guidelines.^23^ Ultimately, BAC scores may offer important and personalized risk stratification information, especially for younger women, without additional time, cost, and radiation.^24^

### Study limitations

First, the retrospective nature of the study does not prove causality. Although attempts at reducing confounding factors using multivariable models were used, residual risk remains. Second, clinical data including outcomes relied on the use of ICD-10 codes from EHR data extraction, which introduces the possibility of misclassification. Also, mortality information only included all-cause mortality, but data on cause-specific mortality including CVD-related death were not available. Third, although EHRs allow for large aggregation of data and study populations with ICD codes for outcome ascertainment, misclassification still occurs. Additionally, while EHRs are becoming increasingly connected across hospital systems, follow-up information is still lost, especially among those who received care in other health systems. Fourth, follow-up varied for women in the study due to use of a strict censoring date, loss to follow-up, and development of events. However, regarding the composite outcome, there were only 146 women with less than 1 year of follow-up, and by the ninth year, there were still 9,804 women with follow-up data available (out of the 16,638 assessed for loss to follow-up; 17,720 total eligible for the composite outcome analyses and 1,082 developed events). Fifth, data on menopausal status were not available. Sixth, most subjects in this study identified as White, making results most applicable to this population. Seventh, our study design adjusted for history of several cardiovascular conditions based on ICD codes, including history of MI, CAD, HF, and PAD. However, we do not have available information on specific CV interventions, such as PCI, coronary artery bypass graft, or valve replacements. Lastly, this study shows the characteristics and outcomes from a single-center, albeit with a large cohort of women. Our ongoing work focuses on assessing the implications of BAC across more diverse populations to increase external validity of this potential screening tool and to identify additional areas to improve risk assessment.

## Conclusions

In this large, retrospective study, both BAC presence and quantity are significantly and independently associated with mortality and CVD outcomes. BAC appears to be especially predictive of CVD risk among younger women. Reporting of BAC was feasible and reliable using an automated AI algorithm, which could facilitate reporting uptake within the radiology community. Further studies are needed to determine the appropriate clinical response to BAC, and whether such a response can improve CVD outcomes in women.

PERSPECTIVES **COMPETENCY IN MEDICAL KNOWLEDGE:** BAC on mammograms can be reliably quantified using a novel software based on an AI algorithm and is independently associated with an increased risk of mortality and CVD. After accounting for traditional cardiovascular risk factors, these associations held true when looking at BAC as a binary, quartile, and continuous variables. BAC was most predictive for mortality and CVD outcomes among younger women (aged 40-59 years), but still independently predictive in women aged 60 to 74 years.

**COMPETENCY IN PATIENT CARE:** Quantification of BAC from normal screening mammograms may improve personalized risk stratification for adverse outcomes and provide patients and clinicians with evidence to guide shared decision-making for preventive measures.

**TRANSLATIONAL OUTLOOK 1:** Our data provide support for the inclusion of BAC findings on mammogram reports.

**TRANSLATIONAL OUTLOOK 2:** Automated quantification tools and reporting methods of BAC will be critical to engagement of radiologists and implementation of reporting.

**TRANSLATIONAL OUTLOOK 3:** While additional studies are needed to determine the appropriate clinical response, the presence of BAC should at the minimum stimulate patient-provider conversations on lifestyle changes to mitigate cardiovascular risk, especially among younger women aged 40 to 59 years.

## Funding support and author disclosures

This research was in part funded by the National Heart, Lung, and Blood Institute T32 HL079891-11. Drs Bui, Nerlekar, Eghtedari, and Daniels have served as consultants to CureMetrix. Drs Mantey and Wang are employees of CureMetrix. All other authors have reported that they have no relationships relevant to the contents of this paper to disclose.
