# Supplementary material for: Automated Breast Arterial Calcification Score Is Associated With Cardiovascular Outcomes and Mortality
Source: JACC Adv. 2024 Sep 27;3(11):101283. doi: 10.1016/j.jacadv.2024.101283 (PMC11470245; doi:10.1016/j.jacadv.2024.101283)
Supplement: Supplemental Figures and Tables [file mmc1.docx]

**Supplemental Table 1. ICD-10 Diagnosis Codes**

| **Diagnosis** | **ICD-10 Code** |
| --- | --- |
| Atherosclerotic Cardiovascular Disease | I25.10 |
| Coronary Artery Disease | I25.1 |
| Cerebrovascular Disease | I60-163, I65-169 |
| Peripheral Arterial Disease | I70.2-I70.8 |
| Myocardial Infarction | I21-I23 |
| Hyperlipidemia | E78 |
| Chronic Kidney Disease | N18 |
| End-Stage Renal Disease | N18.6 |
| Diabetes | E08-E13 |
| Hypertension | I10-I16 |
| Heart Failure | I50 |

**Supplemental Table 2.** **Baseline Participant Characteristics by BAC Score Quartiles**

| **Baseline Characteristics** | **1^st^ Quartile [Score 1-25], n=2,552 (60.4%)** | **2^nd^ Quartile [Score 26-50], n=643 (15.2%)** | **3^rd^ Quartile [Score 51-75], n=509 (12.1%)** | **4^th^ Quartile [Score 76-100], n=519 (12.3%)** | *p* |
| --- | --- | --- | --- | --- | --- |
| **Age**, mean years (SD) | 61.6 (11.3) | 67.8 (9.9) | 71.2 (9.4) | 73.6 (9.2) | <0.001 |
| **Race/Ethnicity** | | | | | |
| Caucasian, No. (%) | 1651 (64.7) | 412 (64.1) | 316 (62) | 238 (45.9) | <0.001 |
| Black/African American, No. (%) | 129 (5.1) | 37 (5.8) | 30 (5.9) | 45 (8.7) | 0.01 |
| Hispanic/Latino, No. (%) | 226 (8.9) | 71 (11.0) | 59 (11.6) | 99 (19.1) | <0.001 |
| Asian/Pacific Islander, No. (%) | 292 (11.4) | 72 (11.2) | 53 (10.4) | 79 (15.2) | 0.06 |
| Other, No. (%) | 254 (10.0) | 51 (7.9) | 51 (10.0) | 58 (11.2) | 0.29 |
| **Diabetes**, No, (%) | 147 (5.8) | 63 (9.8) | 48 (9.4) | 92 (17.7) | <0.001 |
| **Hypertension**, No. (%) | 776 (30.4) | 273 (42.5) | 266 (52.3) | 293 (56.5) | <0.001 |
| **Hyperlipidemia**, No. (%) | 690 (27.0) | 234 (36.4) | 213 (41.9) | 228 (43.9) | <0.001 |
| **History of CVD**, No. (%) | 177 (6.9) | 62 (9.6) | 67 (13.2) | 118 (22.7) | <0.001 |
| **History of CKD**, No. (%) | 56 (2.2) | 23 (3.6) | 19 (3.7) | 36 (6.9) | <0.001 |
| **Current smoking**, No. (%) | 96 (3.8) | 17 (2.6) | 7 (1.4) | 14 (2.7) | 0.03 |
| **Never smokers**, No. (%) | 1284 (50.3) | 279 (43.4) | 237 (46.6) | 246 (47.4) | 0.01 |
| **Systolic blood pressure**, mmHg (IQR) | 126 (19.0) | 130 (16.0) | 132 (19.0) | 132 (18.0) | <0.001 |
| **Total cholesterol**, mg/dL (IQR) | 204 (26.0) | 203 (29.5) | 203 (28.6) | 197 (28.4) | <0.001 |
| **Statin use**, No. (%) | 714 (28.0) | 253 (39.3) | 214 (42.0) | 249 (48.0) | <0.001 |
| **Antihypertensive use**, No. (%) | 618 (24.2) | 235 (36.6) | 207 (40.7) | 253 (48.7) | <0.001 |
| BAC, breast arterial calcification; SD, standard deviation; No, number; CKD, chronic kidney disease; CVD, cardiovascular disease; IQR, interquartile range. | | | | | |

**Supplemental Table 3.** **Imputation and Missing Covariate Data**

| **Variable** | **Values Present** | **Values Imputed** |
| --- | --- | --- |
| Total Cholesterol | 14,210 | 3,882 |
| Low-Density Lipoprotein Cholesterol | 14,274 | 3,818 |
| High-Density Lipoprotein Cholesterol | 14,239 | 3,853 |
| Diastolic Blood Pressure | 16,199 | 1,893 |
| Systolic Blood Pressure | 16,199 | 1,893 |
| Ethnicity | 18,089 | 0 (3 missing, not imputed) |
| Smoking Status | 13,874 | 0 (4,218 “unknown” in categorical variable) |

**Supplemental Table 4. Association of Breast Arterial Calcification Presence and Cardiovascular Outcomes**

|  | **Heart Failure**  **(n=298/17,911)**  **HR (95% CI)** | ***p*** | **Myocardial Infarction**  **(n=83/18,051)**  **HR (95% CI)** | ***p*** | **Stroke**  **(n=259/17,914)**  **HR (95% CI)** | ***p*** |
| --- | --- | --- | --- | --- | --- | --- |
| **Among all participants** | | | | | | |
| Model 1 | **1.51 (1.33, 1.73)** | **<0.001** | 1.11 (0.96, 1.28) | 0.184 | **1.29 (1.13, 1.48)** | **<0.001** |
| Model 2 | **1.46 (1.28, 1.66)** | **<0.001** | 1.10 (0.95, 1.28) | 0.200 | **1.25 (1.09, 1.43)** | **0.001** |
| Model 3 | **1.41 (1.24, 1.61)** | **<0.001** | 1.10 (0.95, 1.27) | 0.214 | **1.22 (1.07, 1.40)** | **0.003** |
| Model 1: unadjusted. Model 2: adjusted for age and race/ethnicity. Model 3: adjusted for age, race/ethnicity, systolic blood pressure, diastolic blood pressure, diabetes, total cholesterol, low-density lipoprotein cholesterol, history of cardiovascular disease, history of chronic kidney disease, and smoking status. HR, hazard ratio; CI, confidence interval. | | | | | | |

**Supplemental Table 5. Association of The BAC Score Quartiles and Clinical Outcomes**

|  | **Heart Failure**  **(n=298/17,911)**  **HR (95% CI)** | ***p*** | **Myocardial Infarction**  **(n=83/18,051)**  **HR (95% CI)** | ***p*** | **Stroke**  **(n=259/17,914)**  **HR (95% CI)** | ***p*** |
| --- | --- | --- | --- | --- | --- | --- |
| BAC negative, n=13,869 | *Referent* | *--* | *Referent* | *--* | *Referent* | *--* |
| 1^st^ Quartile [Score 1-25], n=2552 | **1.17 (1.00, 1.38)** | **0.049** | 1.03 (0.86, 1.23) | 0.767 | 1.06 (0.90, 1.25) | 0.501 |
| 2^nd^ Quartile [Score 26-50], n=643 | **1.38 (1.03, 1.85)** | **0.029** | 1.23 (0.88, 1.71) | 0.232 | **1.35 (1.00, 1.82)** | **0.048** |
| 3^rd^ Quartile [Score 51-75], n=509 | **2.03 (1.49, 2.76)** | **<0.001** | 1.14 (0.79, 1.66) | 0.485 | 1.19 (0.85, 1.67) | 0.322 |
| 4^th^ Quartile [Score 76-100], n=519 | **1.70 (1.23, 2.35)** | **0.001** | 1.12 (0.77, 1.63) | 0.563 | **1.76 (1.27, 2.44)** | **<0.001** |
| All data from the multivariable-adjusted model (Model 3), which adjusted for age, race/ethnicity, systolic blood pressure, diastolic blood pressure, diabetes, total cholesterol, low-density lipoprotein cholesterol, history of cardiovascular disease, history of chronic kidney disease, and smoking status. HR, hazard ratio; CI, confidence interval. | | | | | | |


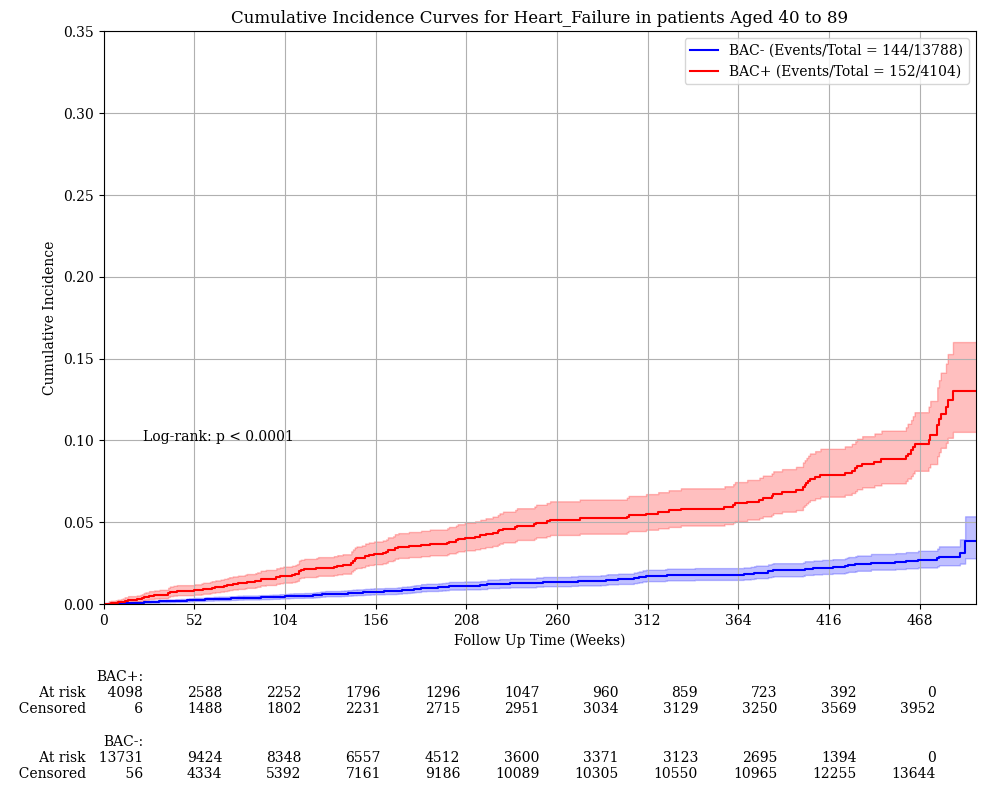

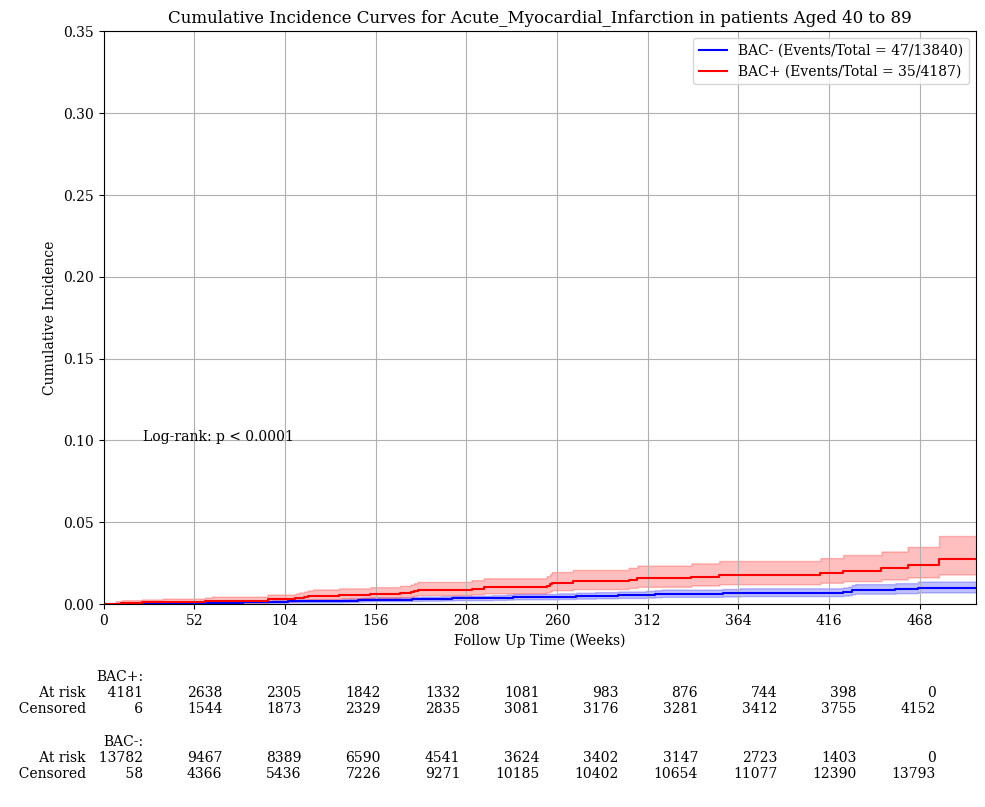


**A**

**B**

**A**


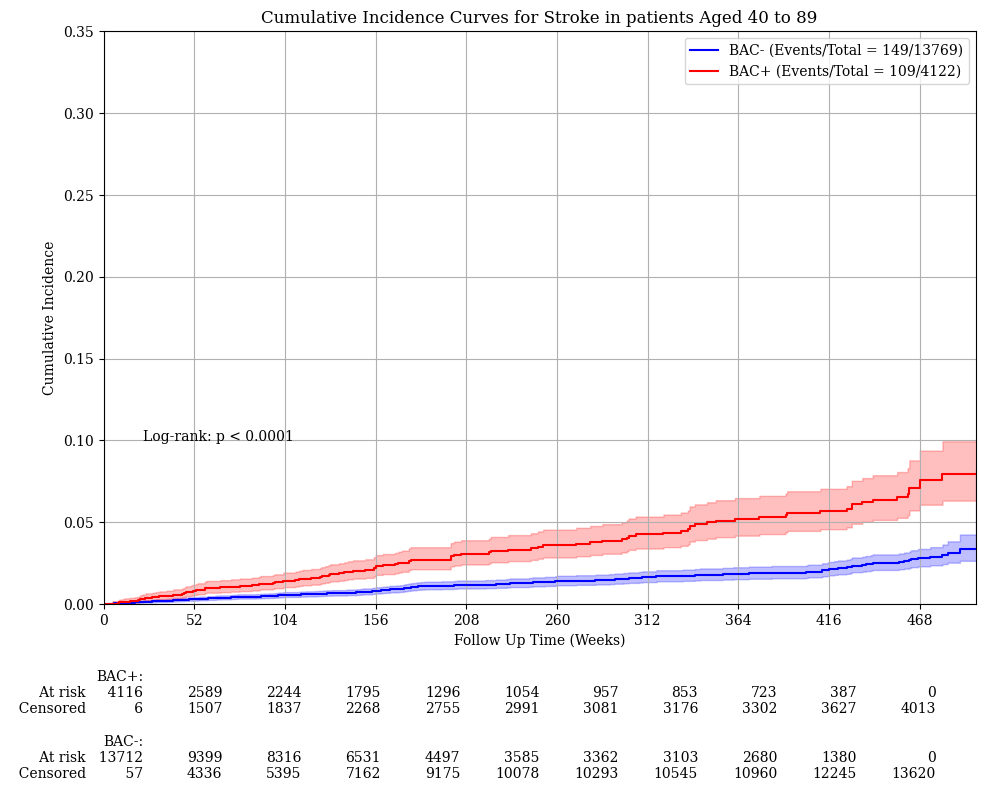


**Supplemental Figure 1. Cumulative Incidence Plots for Acute Myocardial Infarction, Heart Failure, and Stroke by Presence of Breast Arterial Calcification**

Risk for (**A**) acute myocardial infarction, (**B**) heart failure, and (**C**) stroke each significantly varied by the presence of breast arterial calcification (p<0.001) when comparing the unadjusted Cumulative Incidence Curves. BAC indicates breast arterial calcification; BAC+, presence of BAC; BAC-, absence of BAC. Timepoints of 208 weeks and 468 weeks are indicative of approximately 4 years and 9 years, respectively.

**C**


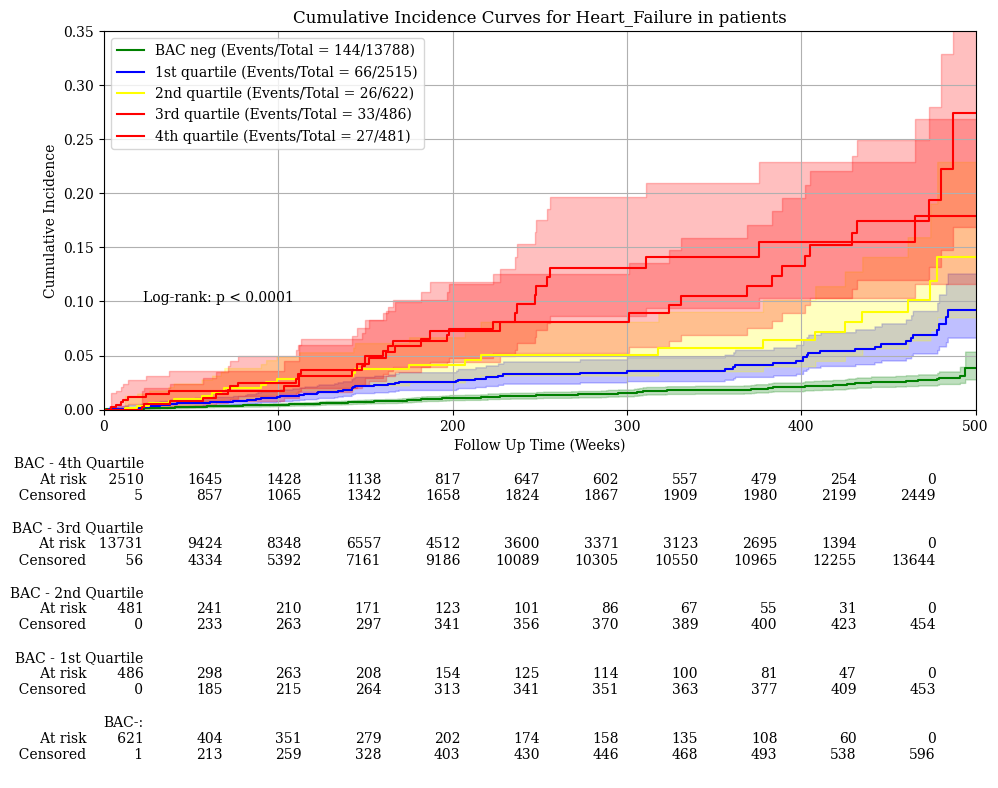

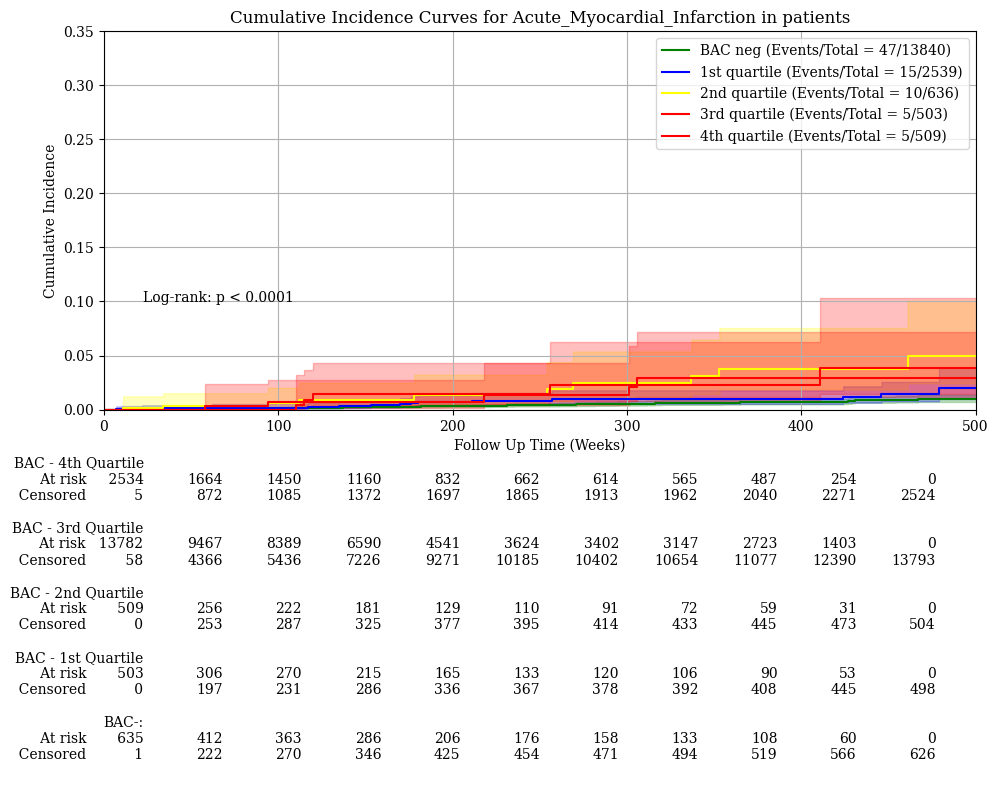


**A**

**B**


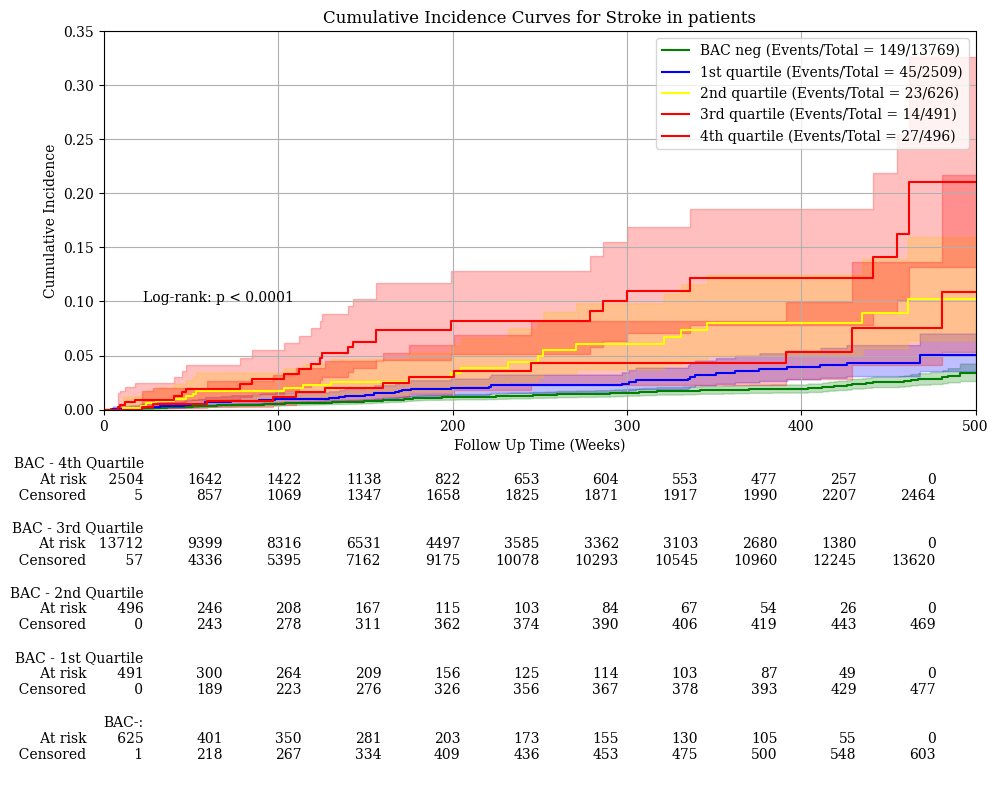


**C**

**Supplemental Figure 2. Cumulative Incidence Plots for Acute Myocardial Infarction, Heart Failure, and Stroke by Breast Arterial Calcification Score Quartiles**

Risk for (**A**) acute myocardial infarction, (**B**) heart failure, and (**C**) stroke each significantly varied by the presence of breast arterial calcification (p<0.001) when comparing the unadjusted cumulative incidence curves. BAC indicates breast arterial calcification; BAC+, presence of BAC; BAC-, absence of BAC. Timepoints of 208 weeks and 468 weeks are indicative of approximately 4 years and 9 years, respectively.
